# Evaluating *TAB2, IKBKB,* and *IKBKG* Gene Polymorphisms and Serum Protein Levels and Their Association with Age-Related Macular Degeneration and Its Treatment Efficiency

**DOI:** 10.3390/medicina60122072

**Published:** 2024-12-16

**Authors:** Alvita Vilkeviciute, Enrika Pileckaite, Akvile Bruzaite, Dzastina Cebatoriene, Greta Gedvilaite-Vaicechauskiene, Loresa Kriauciuniene, Dalia Zaliuniene, Rasa Liutkeviciene

**Affiliations:** 1 Ophthalmology Laboratory, Neuroscience Institute, Lithuanian University of Health Sciences, Medical Academy, Eiveniu 2, LT-50161 Kaunas, Lithuania; enrika.pileckaite@lsmu.lt (E.P.); akvile.bruzaite@lsmu.lt (A.B.); greta.gedvilaite@lsmu.lt (G.G.-V.); loresa.kriauciuniene@lsmu.lt (L.K.); rasa.liutkeviciene@lsmu.lt (R.L.); 2 Department of Ophthalmology, Medical Academy, Lithuanian University of Health Sciences, Eiveniu 2, LT-50161 Kaunas, Lithuania; dzastina.cebatoriene@lsmu.lt (D.C.); dalia.zaliuniene@lsmu.lt (D.Z.)

**Keywords:** age-related macular degeneration, *TAB2* rs237025, *IKBKB* rs13278372, *IKBKG* rs2472395 gene, ELISA, VEGF, treatment

## Abstract

*Background and Objectives*: Age-related macular degeneration (AMD) is the leading cause of blindness, affecting millions worldwide. Its pathogenesis involves the death of the retinal pigment epithelium (RPE), followed by photoreceptor degeneration. Although AMD is multifactorial, various genetic markers are strongly associated with the disease and may serve as biomarkers for evaluating treatment efficacy. This study investigates *TAB2* rs237025, *IKBKB* rs13278372, and *IKBKG* rs2472395 variants and their respective serum protein concentrations in relation to AMD occurrence and exudative AMD treatment response to anti-VEGF treatment. *Materials and Methods:* The case–control study involved 961 individuals, and they were divided into three groups: control, early AMD, and exudative AM patients. Genotyping of selected SNPs were conducted using a real-time polymerase chain reaction method (RT-PCR). Based on the clinical OCT and BCVA data, patients with exudative AMD were categorized into one of two groups: responders and non-responders. The data obtained were analyzed using the “IBM SPSS Statistics 29.0” software program. *Results*: Our study revealed that *TAB2* rs237025 allele A was identified as a risk factor for early and exudative AMD development. The same associations remained only in females with exudative AMD but not in males, suggesting gender-specific pathogenetic pathways in exudative AMD. Analysis of *IKBKB* rs13278372 or serum IKBKB protein associations with early or exudative AMD occurrence in the Lithuanian population revealed no significant associations. On the other hand, we found that each A allele of *IKBKB* rs13278372 was associated with a worse response to anti-VEGF treatment (OR = 0.347; 95% CI: 0.145–0.961; *p* = 0.041). These results suggest a potential marker for future studies evaluating anti-VEGF treatment for exudative AMD patients. *IKBKG* rs2472395 was a protective variant for early AMD in males and for exudative AMD in females only. Also, IKBKG protein concentration was lower in exudative AMD relative to the control group (median (IQR): 0.442 (0.152) vs. 0.538 (0.337), *p* = 0.015). Moreover, exudative AMD patients who carry the GG genotype of *IKBKG* rs2472394 exhibited significantly reduced serum IKBKG concentrations compared to the controls (median (IQR): 0.434 (0.199) vs. 0.603 (0.335), *p* = 0.012), leading to the hypothesis that the *IKBKG* rs2472394 variant might play a role in protein concentration differences and exudative AMD development. *Conclusions*: Our study identified the *TAB2* rs237025 allele A as a significant risk factor for both early and exudative AMD, with gender-specific associations observed in females with exudative AMD, suggesting distinct pathogenetic pathways. While *IKBKB* rs13278372 and serum IKBKB protein levels showed no significant association with AMD development, the A allele of *IKBKB* rs13278372 was associated with a worse response to anti-VEGF treatment, indicating its potential as a marker for treatment outcomes. Additionally, the *IKBKG* rs2472395 variant was found to be protective for early AMD in males and exudative AMD in females, and lower IKBKG protein levels were associated with exudative AMD, particularly in patients with the GG genotype of *IKBKG* rs2472394, suggesting its role in protein concentration and disease progression. These findings highlight genetic markers that may contribute to AMD pathogenesis and treatment response.

## 1. Introduction

Age-related macular degeneration (AMD) is a progressive retinal disease that affects millions of people worldwide. It is one of the main causes of irreversible blindness in industrialized countries and mainly affects people over the age of 60 [1,2]. In Lithuania, as in many other European countries, AMD is a major public health condition. AMD is the main cause of visual impairment in older adults across Europe, and its prevalence is expected to increase due to the aging population. A recent systematic review of AMD in Europe shows that approximately 25% of individuals over the age of 60 are affected by early or intermediate-stage AMD, while about 2.4% experience late-stage AMD [3].

This trend is mirrored by similar figures in Lithuania, where the aging population is likely contributing to a similar increase in AMD cases. Current and future healthcare planning in the region needs to take this growing burden into account. It is estimated that the population with AMD in Europe, including Lithuania, will increase by 15% by 2050 [4].

AMD primarily affects the macula, leading to impaired modifications in the deeper layers of the retina and peripheral blood vessels, culminating in the gradual loss of central vision. Although numerous risk factors have been identified, it is generally recognized that these alone cannot fully explain the onset of AMD [5]. The hallmark of early AMD is the accumulation of drusen, extracellular deposits on the retina that often signal the “dry” form of the disease. Dry AMD, the most common form, can progress to “wet” or neovascular AMD, in which abnormal choroidal neovascularization (CNV) leads to hemorrhages and severe visual impairment. This neovascularization is driven by vascular endothelial growth factor (VEGF), making anti-VEGF therapy the most important treatment for wet AMD [6]. On the other hand, repeated injections usually cannot cure the disease, but they can halt disease progression or delay vision loss. Unfortunately, these injections may not even achieve the desired effect in a number of patients.

Anti-VEGF treatments may slow the progression of the disease but often do not reverse the damage, and many patients do not respond adequately to these therapies. Geographic atrophy, a severe form of dry AMD, does not respond to anti-VEGF therapy and has equally devastating consequences for vision when the fovea is affected [6]. Recent studies suggest that the dry and wet forms of AMD may be distinct diseases, with only the neovascular form being treatable [7,8].

Given the multifactorial nature of AMD, its development is influenced by both environmental and genetic factors. Various genetic markers have been linked to the disease, leading to investigations into the possible genetic contributions to AMD pathogenesis [9]. In particular, new gene therapies have shown promising results in clinical trials and could revolutionize the treatment of wet AMD [10]. Environmental factors, such as smoking and diet or para-inflammation, alongside age-related cellular changes, also play a significant role in disease progression. A recent review by Paliwal et al. (2023) provides a comprehensive overview of the various factors contributing to AMD, offering valuable insights into how these factors can increase disease susceptibility [11]. This review highlights key pathways, including oxidative stress, inflammation, and the role of the immune system, that are critical in the pathogenesis of AMD [11]. By including these insights, our study aims to build upon this understanding, focusing on the genetic aspects and their implications for treatment response.

TGF-beta-activated kinase 1 (MAP3K7)-binding protein 2 (TAB2) is recognized as a MAP3K7/TAK1 activator encoded by TAB2 which is situated on chromosome 6’s long arm (6q25.1). This protein is necessary for the activation of nuclear factor kappa B (NFκB) and MAPK8/JNK molecules by IL-1-1. Additionally, it functions as an adapter connecting MAP3K7 and TRAF6 by forming a kinase complex with TRAF6, MAP3K7, and TAB1. This protein, along with TAB1 and MAP3K7, is also implicated in the signaling cascade that controls osteoclast growth and function through TNFSF11/RANKl, the activating receptor activator of NFκB (TNFRSF11A/RANK) [12]. Moreover, previous studies have confirmed that TAB2 plays an important role in the activation of the NFκB signaling pathway by attaching to polyubiquitin chains [13]. In particular, the EMT and PI3K-AKT signaling pathways, which are linked to tumor cell metastasis and proliferation, are activated by active NFκB [14].

IKKβ (inhibitor of NFκB kinase beta subunit) is a Ser/Thr kinase protein that is encoded by *IKBKB*. This gene is found on chromosome 8’s short arm (8p11.21). The IKKβ protein is the component of the IKK complex, which also includes the key modulator of the IKKα kinase and NF-κB (NEMO/IKKγ). The inhibitor (IκB) in the inhibitor–NF-κB complex is phosphorylated by the IKKβ protein, which results in the inhibitor’s dissociation and NF-κB activation. The proteosome inhibitor is then degraded by K48 ubiquitination of the phosphorylated IκB [15]. Numerous genes involved in cell cycle regulation and apoptosis prevention have their transcription activated by free NF-κB that reaches the nucleus [16]. The *IKBKG* (inhibitor of kappa light polypeptide gene enhancer in B-cells, kinase gamma) gene, located in chromosomal region Xq28, encodes the regulatory subunit of the IKK (inhibitor of kappa B kinase) complex, which is required for activation of the NF-kB signaling pathway [17]. *IKBKG* product stimulates nuclear factor kappa B (NF-κB), a transcription factor that controls the expression of numerous genes in nearly every cell and is implicated in inflammation, innate immunity, cellular stress response, and cell proliferation and survival [18]. The greatest expression level of IKBKG was found in the central nervous system [19]. It was also found that mutations in the *IKBKG* gene, also known as nuclear factor-kappa B (NF-kB) essential modulator (NEMO), are the most frequent single cause of incontinentia pigmenti (IP) in women and anhydrotic ectodermal dysplasia with immunodeficiency (EDA-ID) in men [20].

Considering the importance of these genes in AMD pathology, this study aims to analyze the association of specific polymorphisms in *TAB2* rs237025, *IKBKB* rs13278372, and *IKBKG* rs2472395 with AMD susceptibility and treatment response. Additionally, the correlation between these gene variants and serum protein levels will be evaluated to further understand their role in AMD development and therapy outcomes.

## 2. Materials and Methods

**Figure 1:**
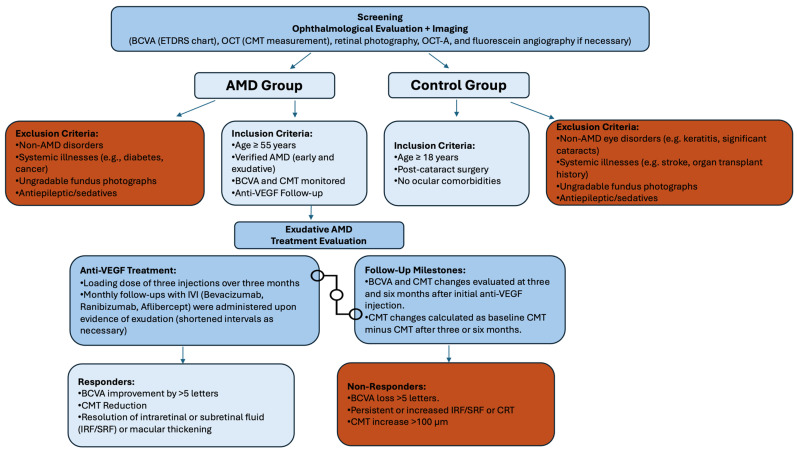
Study inclusion and exclusion criteria.

The study adhered to the guidelines of the Declaration of Helsinki and was given approval from the Kaunas Regional Biomedical Research Ethics Committee of the Lithuanian University of Health Sciences (approval number BE-2-/48). Informed consent was obtained from all participants. Study subjects were admitted to the Ophthalmology Department at the Hospital of the Lithuanian University of Health Sciences between 2014 and 2024, where they underwent comprehensive ophthalmological evaluations. Health information and details of other diseases were collected during examinations by general practitioners and from medical records. This study was conducted using patient samples collected over a 10-year period as part of an ongoing biobank initiative. These samples were not collected exclusively for this study but were also utilized in other research projects. The duration reflects the continuous nature of sample collection and the application of specific inclusion criteria to identify the patients analyzed in this study. Detailed information about the inclusion and exclusion criteria are presented in Figure 1.

The procedures for the ophthalmological evaluation and DNA extraction from peripheral venous blood have been previously reported and published as part of our ongoing project [21].

The genotyping of *TAB2* rs237025 (C_175678357_10), *IKBKB* rs13278372 (C__31336745_10), and *IKBKG* rs2472395 (C__16235530_10) variants was determined according to the manufacturer‘s recommendations using the RT-PCR thermal cycler “StepOne Plus” (Applied Biosystems, Foster City, CA, USA). Serum TAB2 (cat. No. abx251591), IKBKB (cat. No. abx151922), and IKBKG (cat. No. abx258909) protein concentration measurements were conducted applying a Multiskan FC microplate photometer (Thermo Scientific, Waltham, MA, USA) using Abbexa ELISA kits (Abbexa BV, Leiden, The Netherlands).

### 2.1. Selection of SNPs

**Figure 2:**
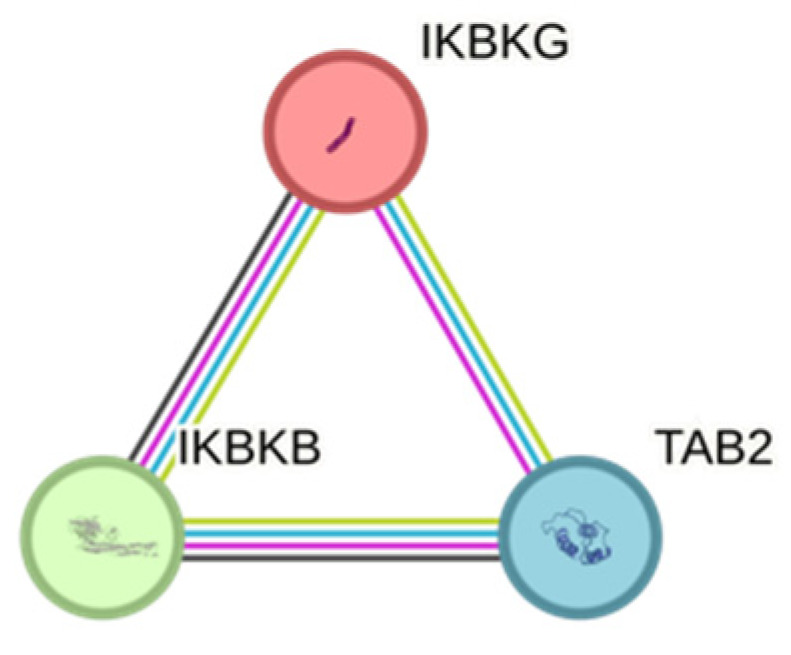
PPI network generated for TAB2, IKBKB, and IKBKG. Types of interaction sources include coexpression (black), experimental data (purple), curation in databases (light blue), and text mining (lime). PPI enrichment *p*-value = 0.000469.

Our study’s single nucleotide polymorphisms (SNPs) were carefully chosen because of their diverse and varied relationships to disease processes. Also, the protein–protein interactions (PPIs) were conducted applying the online STRING v11.0 tool (URL (accessed on 10 September 2024 https://string-db.org) (Figure 2) [22].

We looked closely at previous studies on polymorphisms and how they relate to different illnesses. Following a thorough investigation, we determined which SNPs were most pertinent to investigate in connection with AMD or conditions related to this disease.

The *TAB2* rs237025 risk allele has shown a significant association with type 2 diabetes mellitus (T2DM), a metabolic condition that shares overlapping pathophysiological mechanisms with AMD, such as chronic inflammation and vascular dysfunction [23,24,25]. This suggests that the *TAB2* variant could contribute to the intersection of genetic risk factors influencing both T2DM and AMD pathogenesis.

Furthermore, this variant has been recognized as a significant risk factor for coronary artery disease (CAD) in individuals without diabetes, highlighting its broader relevance to cardiovascular health [26]. Given that CAD shares underlying mechanisms with AMD, such as dyslipidemia, chronic inflammation, and endothelial dysfunction, the *TAB2* rs237025 variant may represent a genetic link connecting these related conditions.

The *IKBKB* rs13278372 and *IKBKG* rs2472395 variants were selected as potential biomarkers for AMD due to their involvement in critical protein–protein interactions shown in Figure 2. IKBKB and IKBKG are integral components of the NF-kB signaling pathway, which regulates the genes associated with immune responses, inflammation, and cell survival [18]. Therefore, dysregulation of this pathway has been implicated in the development of AMD, making these genetic variants particularly relevant for further investigation as biomarkers [27].

Consequently, these findings suggest that *TAB2* rs237025, *IKBKB* rs13278372, and *IKBKG* rs2472395 could serve as important genetic biomarkers for understanding the shared molecular pathways contributing to AMD and related systemic diseases.

### 2.2. Statistical Evaluation

The evaluation of statistical data was performed with SPSS version 29.0 (Statistical Package for the Social Sciences for Windows, Inc., Chicago, IL, USA). Age, BCVA, and CMT were all continuous variables whose normality was evaluated using a Shapiro–Wilk test. Non-normally distributed continuous variables were presented as medians with interquartile ranges (IQR) and analyzed via a Mann–Whitney U test. A Student’s *t* test was used to compare normally distributed data, which were represented as the mean and standard deviation (SD). A Friedman test was applied to evaluate variations in BCVA and CMT values before and after 3 and 6 months of treatment. Statistical significance was set at *p* < 0.05.

Using a Pearson’s chi-square test (χ^2^), categorical data (such as sex and genotype distributions) were compared between groups and presented as absolute values with percentages in parenthesis. The impact of SNPs on patients of early and exudative AMD was examined through binary logistic regression analysis. Assumptions for the binomial regression were confirmed, including a dichotomous dependent variable, independence of observations, and sufficient sample size to ensure reliable estimates. Results were presented as odds ratios (OR) with a 95% confidence interval (CI), corrected for age in the exudative AMD group. Various genetic models were applied, including codominant, dominant, recessive, and overdominant models, while the additive model evaluated the effect of each minor allele on AMD. The Akaike information criterion (AIC) was used to choose the most suitable genetic model; a lower AIC value denotes a better fit. To account for multiple comparisons, a Bonferroni correction was applied, resulting in an adjusted significance threshold of α = 0.0167 (0.05/3, since four SNPs were analyzed).

## 3. Results

The case–control study involved 961 individuals, and they were divided into three groups: control, early AMD, and exudative AM patients. The characteristics of the groups are presented in Table 1. The control group consisted of 337 subjects who matched the gender classification of the early and exudative AMD group. However, the control group subjects were younger than those with exudative AMD (*p* < 0.001), necessitating further analysis adjusted for age.

We examined the genotype and allele frequency distributions of *TAB2* rs237025, *IKBKB* rs13278372, and *IKBKG* rs2472394 in the control and early, exudative AMD groups. The analysis showed that the *TAB2* rs237025 A allele is statistically significantly more frequent in exudative AMD patients than in control subjects (47.2% vs. 41.2%, *p* = 0.026) (Table 2). Unfortunately, this result did not survive the strict Bonferroni correction.

Also, results showed that the *IKBKG* rs2472394 distribution of CC, AC, and AA genotypes is statistically significantly varied in early AMD patients evaluated against the control group (89.3%, 9.3%, and 1.4% vs. 84.3%, 11.0%, and 4.7%, *p* = 0.047) (Table 2). Although, this result did not withstand the Bonferroni correction. When compared to the control group, the A allele of *IKBKG* rs2472394 is statistically significantly less common in patients with early AMD than in controls (6.1% vs. 10.2%, *p* = 0.008) (Table 2).

To assess the impact of certain gene polymorphisms on the onset of early AMD, we conducted a binomial logistic regression analysis (Table 3). The findings revealed that the AA + AG genotypes of *TAB2* rs237025 increase the odds of developing early AMD by 1.4-fold under the dominant model (OR = 1.442, 95% CI: 1.019–2.040, *p* = 0.039). Still, this result did not reach the Bonferroni-corrected significance level.

Analysis of *IKBKG* rs2472394 showed that compared to the CC genotype, the AA genotype decreases the odds of developing early AMD by 3.5-fold under the codominant model (OR = 0.283, 95% CI: 0.093–0.857, *p* = 0.026). Under the recessive model, the *IKBKG* AA genotype reduces these odds by 3.5-fold (OR = 0.290, 95% CI: 0.096–0.877, *p* = 0.028). Each A allele decreased the odds of early AMD incidence by 1.5-fold under the additive model (OR = 0.648, 95% CI: 0.445–0.944, *p* = 0.024). These findings fell short of statistical significance when we used the Bonferroni-adjusted significance criterion.

Binomial logistic regression analysis was also performed in the control group and patients with exudative AMD (Table 4).

The findings showed that the *TAB2* rs237025 AA genotype compared with the GG genotype enhances the possibility of exudative AMD by 1.7-fold (OR = 1.650, 95% CI: 1.037–2.624, *p* = 0.035), while AA + AG genotypes increase these odds by 1.4-fold under the dominant model (OR = 1.413, 95% CI: 1.003–1.991, *p* = 0.048). Also, each A allele enhances these odds by 1.5-fold under the additive model (OR = 1.291, 95% CI: 1.027–1.622, *p* = 0.029). Unfortunately, these findings fell short of the Bonferroni-corrected significance threshold (Table 4).

Given that sex plays a distinct role in the pathophysiology of AMD, we conducted SNP analysis on males and females independently using the genotyping data (Table 5).

In the group of exudative AMD females, the *TAB2* rs237025 A allele was statistically significantly more prevalent compared to the controls (49.1% vs. 41.7%, *p* = 0.028) (Table 5). Unfortunately, the Bonferroni correction was not able to preserve this finding.

Logistic regression analysis in women with early AMD did not reveal statistically significant results (Table 6).

However, the findings revealed that under the codominant model, the AA genotype of *TAB2* rs237025 increases the odds of exudative AMD in women by 1.8-fold (OR = 1.826, 95% CI: 1.003–3.324, *p* = 0.049), while the additive model shows that each A allele increases these odds by 1.4-fold (OR = 1.364, 95% CI: 1.014–1.835, *p* = 0.040). Unfortunately, the results remained insignificant after the Bonferroni correction (Table 7).

Analysis of *IKBKG* rs2472394 showed that the AA genotype decreases the possibility of exudative AMD occurrence in females by 3.9-fold under the codominant model (OR = 0.259, 95% CI: 0.072–0.933, *p* = 0.039), while under the recessive model, the AA genotype of the *IKBKG* rs2472394 decreases these odds by 3.9-fold (OR = 0.254, 95% CI: 0.071–0.913, *p* = 0.036). These results did not attain statistical significance when we used the Bonferroni-modified significance criterion (Table 7).

Additionally examined were the frequency distributions of the genotypes and alleles of *TAB2* rs237025, *IKBKB* rs13278372, and *IKBKG* rs2472394 in men in the early and exudative AMD and control groups (Table 8). The analysis showed that when compared to control males, the A allele of *IKBKG* rs2472394 is statistically significantly less common in males with early AMD than control males (5.4% vs. 8.8%, *p* = 0.032) (Table 8). However, after Bonferroni correction, the result for early AMD is no longer statistically significant.

After applying the Bonferroni correction for multiple comparisons, some results that were initially statistically significant (*p* < 0.05) did not remain significant. This adjustment is highly conservative and aims to control for Type I errors, particularly given the number of comparisons conducted in this study. However, it also increases the risk of Type II errors, potentially masking true associations. It means that while several associations did not remain statistically significant after adjustment, the unadjusted *p*-values suggest trends that may still hold biological or clinical relevance. These findings should therefore be interpreted with caution, and further validation in independent cohorts is recommended to confirm these trends.

The logistic regression analysis in males with early or exudative AMD did not reveal significant results as well (Table 9 and Table 10).

### 3.1. Exudative AMD Treatment Response and SNPs

A total of 108 patients with exudative AMD had their response to therapy evaluated. The population’s clinical and demographic characteristics are provided in Table 11. The proportion of respondents and non-responders did not differ in terms of age or gender.

We noticed that the central macular thickness (CMT) was lower in responders compared to the non-responders before the treatment (298 (101.75) vs. 410 (174.5), *p* < 0.001), and the median visual acuity (VA) was higher in the responder group compared to the non-responders but only after 6 months of treatment (0.35 (0.35) vs. 0.21 (0.29), *p* = 0.029) (Table 11).

In contrast, a Friedman test was used to analyze in comparison with the VA and CMT data before and after treatment; the VA did not differ after 6 months of therapy (0.35 (0.25) vs. 0.35 (0.35), *p* = 0.048) in responders, while CMT was improved after the treatment (298 (101.75) vs. 273 (87.0), *p* < 0.001). The CMT parameter was also decreased after the treatment in the non-responders’ group (410 (174.5) vs. 279 (109.25), *p* = 0.002) (Table 11).

The investigation of the relationship between all SNPs and the responsiveness to anti-VEGF injections was conducted using binomial logistic regression analysis (Table 12). We were unable to perform the analysis for *IKBKG* as all the subjects had CC genotypes. The results showed that each A allele of *IKBKB* rs13278372 was indicated with a worse response to anti-VEGF therapy under the additive genetic model (OR = 0.347; 95% CI: 0.145–0.961; *p* = 0.041).

### 3.2. Serum Protein Level and SNP Associations with AMD

**Figure 3:**
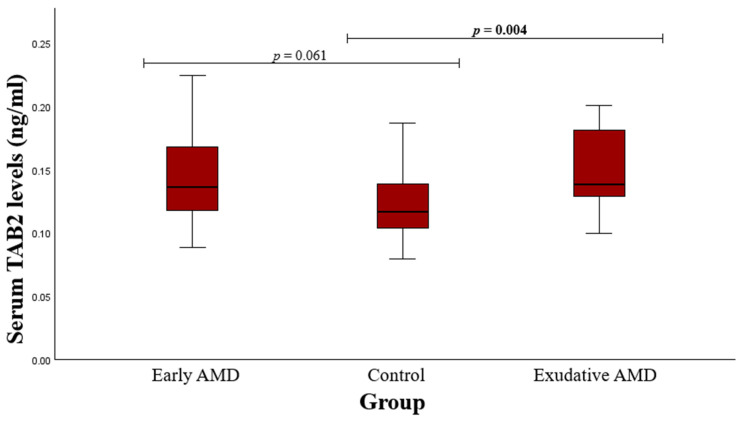
Serum TAB2 levels in patients with early and exudative AMD and the control group; a Mann–Whitney U test was used; *p* values in bold are statistically significant.

The serum levels of TAB2 protein were also measured in early and exudative AMD patients and control subjects. Serum TAB2 levels were found to be higher in the patients with exudative AMD than the healthy individuals (median (IQR): 0.138 (0.055) vs. 0.117 (0.037), *p* = 0.004). Nevertheless, there was no statistically significant difference between the control group and the early AMD group (median (IQR): 0.137 (0.062) vs. 0.117 (0.037), *p* = 0.061). The findings are presented in Figure 3.

**Figure 4:**
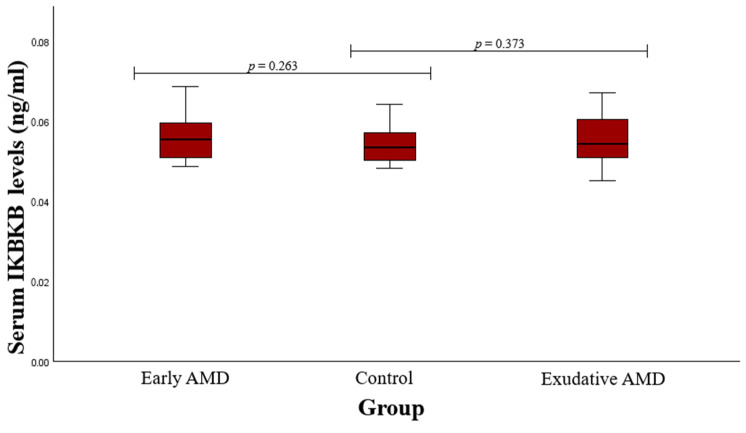
Serum IKBKB levels in patients with early and exudative AMD and the control group; a Mann–Whitney U test was used.

Serum IKBKB concentrations were assessed in patients with early and exudative AMD as well as in control subjects. A statistically significant deviation between patients who developed early AMD and the controls (median (IQR): 0.056 (0.009) vs. 0.054 (0.008), *p* = 0.263) or between patients with exudative AMD and controls was not found (median (IQR): 0.054 (0.010) vs. 0.054 (0.008), *p* = 0.373). These findings are presented in Figure 4.

**Figure 5:**
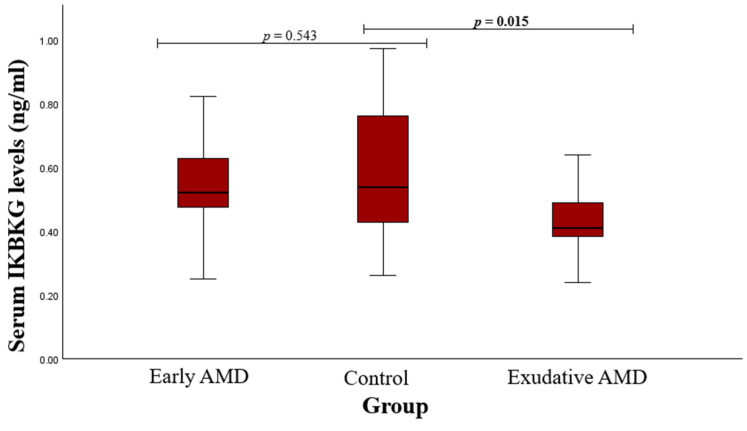
Serum IKBKG levels in patients with early and exudative AMD and the control group; a Mann–Whitney U test was used; *p* values in bold are statistically significant.

IKBKG protein concentrations were measured in patients with early and exudative AMD, along with healthy subjects. Statistically significant deviations were not found between the early AMD group and the control group (median (IQR): 0.523 (0.174) vs. 0.538 (0.337), *p* = 0.543). Results showed that IKBKG protein concentration was lower in the exudative AMD group compared to controls (median (IQR): 0.442 (0.152) vs. 0.538 (0.337), *p* = 0.015). The outcomes are displayed in Figure 5.

**Figure 6:**
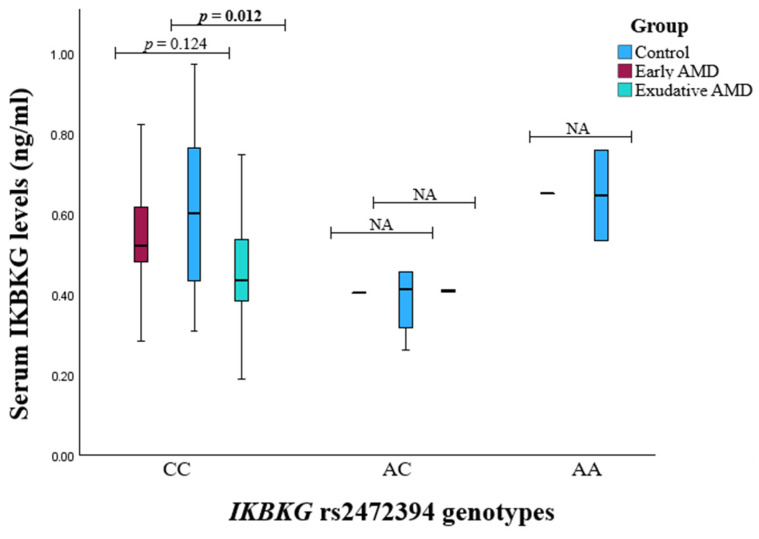
Serum IKBKG levels stratified by *IKBKG* genotypes across the control, early, and exudative AMD study groups; *p* values in bold are statistically significant.

The serum levels of TAB2, IKBKB, and IKBKG were analyzed across different genotypes for specific SNPs. No significant associations were found between TAB2 and IKBKB levels and their genotypes with the development of early or exudative AMD. However, patients who developed exudative AMD with the GG genotype of *IKBKG* rs2472394 had significantly reduced serum IKBKG concentrations compared to the controls (median (IQR): 0.434 (0.199) vs. 0.603 (0.335), *p* = 0.012) (Figure 6).

## 4. Discussion

Our study was conducted on 281 patients with early AMD, 343 patients with exudative AMD, and 337 control subjects. We aimed to analyze *TAB2* rs237025, *IKBKB* rs13278372, and *IKBKG* rs2472394 and respective serum protein concentration associations with early and exudative AMD development and exudative AMD treatment response. According to the scientific literature, such associations were investigated for the first time.

### 4.1. TAB2 rs237025

TAB2 is a TAK1 activator that stimulates NFκB and MAPK8/JNK signaling pathways in an IL-1-dependent manner [12]. The extended expression of TAK1, which can trigger the TAB2 molecule, is linked to non-small cell lung cancer [28]. Furthermore, it has been demonstrated that the TAK1–TAB2–TAB3 signaling pathway is crucial for the bone loss brought on by carcinoma [29]. The TAB2 polypeptide is additionally identified to facilitate the activity of EMT and the PI3K-AKT signaling axis, which are related to the growth and spread of tumor cells via indirectly activating NFκB [14]. In comparison, a current study analyzing serum TAB2 protein concentration showed elevated TAB2 levels in the exudative AMD patients compared to controls (median (IQR): 0.138 (0.055) vs. 0.117 (0.037), *p* = 0.004).

A previous study conducted by Noso et al. (2007) revealed that the frequency of genotypes with the G allele of the *TAB2* rs237025 variant was significantly enhanced in patients with type 2 diabetes [OR, 1.46; 95% (CI) 1.08–1.93; *p* = 0.01] [24]. Later, these results were confirmed by Pu et al. (2012), who showed the rs237025 G allele was significantly more frequent in the T2DM group than in controls as well (0.334 vs. 0.282, *p* = 0.017). Meanwhile, the G allele carriers had higher odds of T2DM under the dominant (*p* = 0.002; OR, 1.525; 95% CI, 1.169–1.989) and overdominant [*p* = 0.001; (OR), 1.563; 95% (CI) 1.189–2.053] genetic models [23]. Another study excluded the patients who had the diabetes and showed that G allele at *TAB2* rs237025 is also linked to higher odds of CAD under the additive (OR = 1.88, 95% CI, 1.31–2.71, *p* = 6.37 × 10^−4^), dominant (OR = 2.11, 95% CI, 1.32–3.35, *p* = 1.69 × 10^−3^), and recessive (2.63 (1.16–4.78), *p* = 0.02) models [26].

Our study revealed that the A allele is more frequent than the G allele in the Lithuanian population, so further results showed that the less frequent allele A at *TAB2* rs237025 enhances the odds of early AMD development by 1.4-fold only under the dominant model (*p* = 0.039).

Patients with exudative AMD had a statistically significant higher frequency of the same A allele than the control group (*p* = 0.026). The odds of developing exudative AMD are increased by 1.7-fold under the codominant model (*p* = 0.035) and by 1.4-fold under the dominant model (*p* = 0.048) when the AA genotype of *TAB2* rs237025 is compared to the GG genotype. Additionally, the additive model shows that these odds are increased by 1.5-fold for every A allele (*p* = 0.029).

In the group of exudative AMD females, the *TAB2* rs237025 A allele is statistically significantly more prevalent than in the control females (*p* = 0.028). Further analysis revealed that under the codominant model, the AA genotype of *TAB2* rs237025 enlarges the likelihood of exudative AMD in women by 1.8-fold (*p* = 0.049), while the additive model shows that each A allele increases these odds by 1.4-fold (*p* = 0.040). However, the results remained insignificant after the Bonferroni correction. Moreover, no significant results were observed in males suggesting gender-specific pathogenetic pathways in exudative AMD.

### 4.2. IKBKB rs13278372

The IKKβ protein, encoded by the *IKBKB* gene, plays a key role in activating the NFκB pathway through phosphorylation of IκB [15]. This activation has been linked to various cancers, including lung adenocarcinoma, melanoma, pancreatic cancer, and gastric cancer. Research has shown that IKKβ promotes lung tumor development by converting lung alveolar epithelial cells into cancerous cells, while its chemical inhibition suppresses tumor growth [30]. In melanoma, IKKβ has a dual role: its absence inhibits malignancy in melanocytes but impairs the phagocytic function of myeloid cells, which is crucial for eliminating cancer cells [31]. Additionally, IKKβ contributes to the possibility of acute pancreatitis by stimulating the infiltration of leukocytes in the pancreas, while its elimination reduces pancreatic cancer risks [32,33].

A study found that the *IKBKB* rs2272736 polymorphism is associated with survival in gastric cancer patients. Specifically, individuals with the G allele had significantly longer survival than those with the A allele [34].

The *IKBKB* rs13278372 variant was analyzed only in pituitary adenoma patients, but no significant associations were found [35].

Unfortunately, our study did not reveal *IKBKB* rs13278372 or serum IKBKB protein associations with early or exudative AMD occurrence in the Lithuanian population either. On the other hand, our analysis did reveal that each A allele of *IKBKB* rs13278372 was linked to a worse reaction to anti-VEGF therapy under the additive genetic model (*p* = 0.041). These results suggest a potential marker for future studies on the evaluation of anti-VEGF treatment for exudative AMD patients.

### 4.3. IKBKG rs2472395

The IKBKG protein interacts with IKK-alpha and IKK-beta enzymes to activate NF-κB, which then moves into the nucleus and regulates the expression of genes involved in immune and inflammatory reactions. NF-κB also plays a role in protecting cells from apoptosis [36]. Dysregulation of this pathway is linked to many diseases [18]. Mutations in IKBKG are associated with the condition incontinentia pigmenti (IP), but if no mutations are detected, an IP diagnosis is excluded [37]. The expression of IKBKG mutations is highly variable, even among related individuals with the same mutation, which may be due to skewed X chromosome inactivation [38]. This phenomenon occurs in women with IP, where the X chromosome with the mutated *IKBKG* allele is preferentially inactivated [39,40]. Additionally, the variability in IP’s phenotype may also result from the broad, pleiotropic effects of NF-κB [41].

In a study by Wang et al. on 42 patients with incontinentia pigmenti (IP), those with pathogenic *IKBKG* variants showed higher rates of hair (50% vs. 14%), dental (70% vs. 21%), and eye abnormalities (45% vs. 29%) compared to those without the variants. However, they were less likely to have central nervous system (CNS) abnormalities (20% vs. 35%) [42]. This highlights the need for detailed phenotypic and genotypic assessments. Previous research shows IP’s clinical presentation ranges from mild skin lesions to severe CNS abnormalities like strokes [40]. Studies suggest that severe CNS anomalies are linked to random X chromosome inactivation, while mild cases show skewed inactivation [43]. Additionally, the type of alteration such as deletion vs. point mutation does not associate with disease severity [44], likely due to the complex role of the NEMO/IKKgamma protein in gene regulation, which may explain the broad spectrum of IP symptoms [45].

Our results showed that the genotypes CC, AC, and AA of the *IKBKG* rs2472394 variant distribution statistically significantly deviate in early AMD patients compared to the controls (89.3%, 9.3%, and 1.4% vs. 84.3%, 11.0% and 4.7%, *p* = 0.047), resulting in the A allele of *IKBKG* rs2472394 being statistically significantly less common in patients with early AMD than in controls (6.1% vs. 10.2%, *p* = 0.008). Moreover, the A allele decreases the odds of developing early AMD by 3.5-fold under the codominant (*p* = 0.026), by 3.5-fold under the recessive (*p* = 0.028), and by 1.5-fold under the additive (*p* = 0.024) models. The same findings remained in males: the A allele of *IKBKG* rs2472394 is statistically significantly less common in males with early AMD than control males (5.4% vs. 8.8%, *p* = 0.032) but not in females.

In exudative AMD, analysis of *IKBKG* rs2472394 showed that the AA genotype decreases the possibility of exudative AMD occurrence by 3.9-fold under the codominant (*p* = 0.039) and recessive (*p* = 0.036) models only in females but not in males.

However, after Bonferroni correction, the result for early AMD is no longer statistically significant. As previously mentioned, these findings should therefore be interpreted with caution, and further validation in independent cohorts is recommended to confirm these trends.

Serum IKBKG levels were also evaluated, and the results showed that IKBKG protein concentration was lower in patients with exudative AMD compared to controls (median (IQR): 0.442 (0.152) vs. 0.538 (0.337), *p* = 0.015). Moreover, serum IKBKG concentrations were considerably lower in exudative AMD patients with the GG genotype of IKBKG rs2472394 than in controls (median (IQR): 0.434 (0.199) vs. 0.603 (0.335), *p* = 0.012), leading to the hypothesis that the *IKBKG* rs2472394 variant might play a role in protein concentration differences and exudative AMD development, but additional investigations are needed to confirm the mentioned findings.

Our results contribute to the expanding body of knowledge on the genetic basis of AMD. Established risk variants in genes such as *CFH* and *ARMS2* have consistently been shown to play a pivotal role in AMD pathogenesis by influencing inflammation and complement activation in various populations as reviewed by Ismail, F et al. (2024). Comparatively, the SNPs analyzed in this study also appear to be associated with pathways implicated in AMD development, including inflammation and oxidative stress [46].

For instance, variants in the CFH gene are known to disrupt complement regulation, leading to chronic inflammation in the retina [47], while ARMS2 variants have been linked to mitochondrial dysfunction and oxidative stress [48,49].

Similar to these mechanisms, the SNPs in our study may influence AMD pathogenesis through the NF-κB signaling pathway, which regulates inflammatory and oxidative processes.

The SNPs analyzed in this study were associated not only with the development of AMD but also with the response to treatment, highlighting their potential dual role in disease progression and management. Genetic variations in loci related to the NF-κB signaling pathway such as *TAB2* rs237025, *IKBKB* rs13278372, and *IKBKG* rs2472395 may modulate inflammatory and oxidative stress pathways, both critical in AMD pathogenesis and therapeutic response. For instance, altered NF-κB activity could influence retinal inflammation, thereby affecting the efficacy of treatments such as anti-VEGF therapy. Therefore, clinical trials investigating NF-κB inhibitors in AMD explore the potential therapeutic benefits of targeting NF-κB, a transcription factor pivotal in regulating inflammation [27].

These findings underscore the importance of integrating genetic data with treatment outcomes to improve personalized medicine strategies. Future studies should aim to validate these associations and directly compare the magnitude and functional relevance of the SNPs with the established risk variants to clarify their relative contributions to AMD. Also, further studies are needed to explore the functional consequences of studied SNPs in the context of both disease progression and therapeutic response, particularly focusing on pathways like NF-κB that link genetic variation to clinical outcomes.

## 5. Conclusions

In conclusion, our study identified the *TAB2* rs237025 allele A as a significant risk factor for both early and exudative AMD, with gender-specific associations observed in females with exudative AMD, suggesting distinct pathogenetic pathways. While *IKBKB* rs13278372 and serum IKBKB protein levels showed no significant association with AMD development, the existence of the A allele of *IKBKB* rs13278372 was related to a worse response to anti-VEGF treatment, indicating its potential as a marker for treatment outcomes. Additionally, the *IKBKG* rs2472395 variant was found to be protective for early AMD in males and exudative AMD in females, and lower IKBKG protein levels were associated with exudative AMD, particularly in patients with the GG genotype of *IKBKG* rs2472394, suggesting its role in protein concentration and disease progression. These findings highlight several genetic markers that may contribute to AMD pathogenesis and treatment response, suggesting further investigation.

However, a key limitation of this study is its focus on a single Lithuanian population. While this reduces genetic heterogeneity and enhances internal validity, it limits generalizability to populations with different genetic, environmental, and lifestyle backgrounds. Variations in allele frequencies, linkage disequilibrium patterns, or gene–environment interactions in other populations may influence the observed associations. Replication in larger, ethnically diverse cohorts is essential to confirm these findings, assess their consistency, and uncover population-specific factors, ensuring the relevance of this research to global precision medicine.

## Figures and Tables

**Table 1:** Demographic data of the study.

Characteristics		Groups			*p*-Value
		Control	Early AMD	Exudative AMD	
Gender	Females, n (%)	218 (64.7)	195 (69.4)	223 (65.0)	0.217 * 0.929 **
	Males, n (%)	119 (35.3)	86 (30.6)	120 (35.0)	
Age, Median (IQR)		72 (11)	73.5 (13)	77 (10)	0.056 * <0.001 **

*p*—significance level; IQR—interquartile range; * early AMD vs. control group; ** exudative AMD vs. control group; *p* values in bold are statistically significant.

**Table 2:** Genotype and allele frequencies of *TAB2* rs237025, *IKBKB* rs13278372, and *IKBKG* rs2472394 in patients with early and exudative AMD and controls.

Genotype/Allele	Groups			*p*-Value *	*p*-Value **
	Control, n (%) N = 337	Early AMD, n (%) N = 281	Exudative AMD, n (%) N = 343		
*TAB2* rs237025					
GG	116 (34.4)	75 (26.7)	92 (26.8)	0.111	0.072
AG	164 (48.7)	150 (53.4)	178 (51.9)		
AA	57 (16.9)	56 (19.9)	73 (21.3)		
G	396 (58.8)	300 (53.4)	362 (52.8)	0.058	**0.026**
A	278 (41.2)	262 (46.6)	324 (47.2)		
*IKBKB* rs13278372					
CC	274 (81.3)	226 (80.4)	270 (78.7)	0.834	0.662
AC	58 (17.2)	52 (18.5)	66 (19.2)		
AA	5 (1.5)	3 (1.1)	7 (2.0)		
C	606 (89.9)	504 (89.7)	606 (88.3)	0.894	0.352
A	68 (10.1)	58 (10.3)	80 (11.7)		
*IKBKG* rs2472394					
CC	284 (84.3)	251 (89.3)	297 (86.6)	**0.047**	0.441
AC	37 (11.0)	26 (9.3)	36 (10.5)		
AA	16 (4.7)	4 (1.4)	10 (2.9)		
C	605 (89.8)	528 (93.9)	630 (91.8)	**0.008**	0.186
A	69 (10.2)	34 (6.1)	56 (8.2)		

*p*—significance level and Bonferroni-corrected significance level when *p* = 0.05/3; AMD—age-related macular degeneration; * early AMD vs. control group; ** exudative AMD vs. control group; *p* values in bold are statistically significant.

**Table 3:** Binomial logistic regression analysis of *TAB2* rs237025, *IKBKB* rs13278372, and *IKBKG* rs2472394 in the control and patients with early AMD groups.

Model	Genotype/Allele	OR (95% CI)	*p*-Value	AIC
*TAB2* rs237025				
Codominant	AG vs. GG AA vs. GG	1.415 (0.982–2.038) 1.520 (0.950–2.430)	0.063 0.081	851.227
Dominant	AA + AG vs. GG	1.442 (1.019–2.040)	**0.039**	849.333
Recessive	AA vs. GG + AG	1.223 (0.813–1.839)	0.335	852.719
Overdominant	AG vs. GG + AA	1.208 (0.880–1.659)	0.243	852.284
Additive	A	1.254 (0.996–1.580)	0.054	849.926
*IKBKB* rs13278372				
Codominant	AC vs. CC AA vs. CC	1.087 (0.719–1.644) 0.727 (0.127–3.077)	0.693 0.665	855.282
Dominant	AA + AC vs. CC	1.058 (0.708–1.583)	0.782	853.572
Recessive	AA vs. CC + AC	0.717 (0.170–3.025)	0.650	853.438
Overdominant	AC vs. CC + AA	1.092 (0.723–1.651)	0.675	853.473
Additive	A	1.025 (0.712–1.476)	0.895	853.631
*IKBKG* rs2472394				
Codominant	AC vs. CC AA vs. CC	0.795 (0.468–1.350) 0.283 (0.093–0.857)	0.396 **0.026**	849.053
Dominant	AA + AC vs. CC	0.640 (0.397–1.034)	0.068	850.235
Recessive	AA vs. CC + AC	0.290 (0.096–0.877)	**0.028**	847.780
Overdominant	AC vs. CC + AA	0.827 (0.487–1.403)	0.480	853.147
Additive	A	0.648 (0.445–0.944)	**0.024**	848.204

OR—odds ratio; CI—confidence interval; *p*—significance level, Bonferroni-corrected significance level *p* = 0.05/3; AIC—Akaike information criteria; *p* values in bold are statistically significant.

**Table 4:** Binomial logistic regression analysis of *TAB2* rs237025, *IKBKB* rs13278372, and *IKBKG* rs2472394 in the control and patients with exudative AMD groups.

Model	Genotype/Allele	OR * (95% CI)	*p*-Value	AIC
*TAB2* rs237025				
Codominant	AG vs. GG AA vs. GG	1.334 (0.929–1.915) 1.650 (1.037–2.624)	0.118 **0.035**	880.643
Dominant	AA + AG vs. GG	1.413 (1.003–1.991)	**0.048**	879.594
Recessive	AA vs. GG + AG	1.378 (0.919–2.067)	0.121	881.094
Overdominant	AG vs. GG + AA	1.102 (0.805–1.510)	0.544	883.146
Additive	A	1.291 (1.027–1.622)	**0.029**	878.697
*IKBKB* rs13278372				
Codominant	AC vs. CC AA vs. CC	1.070 (0.712–1.607) 1.155 (0.348–3.836)	0.746 0.813	885.363
Dominant	AA + AC vs. CC	1.077 (0.727–1.595)	0.712	883.378
Recessive	AA vs. CC + AC	1.141 (0.344–3.777)	0.830	883.468
Overdominant	AC vs. CC + AA	1.066 (0.710–1.601)	0.757	883.419
Additive	A	1.071 (0.757–1.515)	0.697	883.363
*IKBKG* rs2472394				
Codominant	AC vs. CC AA vs. CC	0.968 (0.579–1.616) 0.476 (0.206–1.103)	0.900 0.084	882.432
Dominant	AA + AC vs. CC	0.803 (0.513–1.257)	0.337	882.589
Recessive	AA vs. CC + AC	0.478 (0.207–1.105)	0.084	880.447
Overdominant	AC vs. CC + AA	0.998 (0.599–1.664)	0.994	883.515
Additive	A	0.787 (0.565–1.097)	0.158	881.506

* OR adjusted for age in exudative AMD group; OR—odds ratio; CI—confidence interval; *p*—significance level, Bonferroni-corrected significance level *p* = 0.05/3; AIC—Akaike information criteria; *p* values in bold are statistically significant.

**Table 5:** Genotype and allele frequencies of *TAB2* rs237025, *IKBKB* rs13278372, and *IKBKG* rs2472394 in early and exudative AMD and control groups between females.

Genotype/Allele	Groups			*p*-Value *	*p*-Value **
	Control, n (%)	Early AMD, n (%)	Exudative AMD, n (%)		
*TAB2* rs237025					
GG	74 (33.9)	51 (26.2)	53 (23.8)	0.220	0.055
AG	106 (48.6)	104 (53.3)	121 (54.3)		
AA	38 (17.4)	40 (20.5)	49 (22.0)		
G	254 (58.3)	206 (52.8)	227 (50.9)	0.116	**0.028**
A	182 (41.7)	184 (47.2)	219 (49.1)		
*IKBKB* rs13278372					
CC	172 (78.9)	159 (81.5)	170 (76.2)	0.556	0.796
AC	41 (18.8)	34 (17.4)	47 (21.1)		
AA	5 (2.3)	2 (1.0)	6 (2.7)		
C	385 (88.3)	352 (90.3)	387 (86.8)	0.366	0.491
A	51 (11.7)	38 (9.7)	59 (13.2)		
*IKBKG* rs2472394					
CC	178 (81.7)	168 (86.2)	184 (82.5)	0.079	0.470
AC	32 (14.7)	26 (13.3)	35 (15.7)		
AA	8 (3.7)	1 (0.5)	4 (1.8)		
C	388 (89.0)	362 (92.8)	403 (90.4)	0.057	0.504
A	48 (11.0)	28 (7.2)	43 (9.6)		

*p*—significance level and Bonferroni-corrected significance level when *p* = 0.05/4; AMD—age-related macular degeneration; * early AMD vs. control group; ** exudative AMD vs. control group; *p* values in bold are statistically significant.

**Table 6:** Binomial logistic regression analysis of early AMD and control groups in females.

Model	Genotype/Allele	OR (95% CI)	*p*-Value	AIC
*TAB2* rs237025				
Codominant	AG vs. GG AA vs. GG	1.424 (0.910–2.227) 1.527 (0.864–2.699)	0.122 0.145	572.213
Dominant	AA + AG vs. GG	1.451 (0.949–2.219)	0.086	570.283
Recessive	AA vs. GG + AG	1.222 (0.746–2.002)	0.425	572.621
Overdominant	AG vs. GG + AA	1.208 (0.820–1.778)	0.339	572.344
Additive	A	1.256 (0.949–1.663)	0.112	570.711
*IKBKB* rs13278372				
Codominant	AC vs. CC AA vs. CC	0.897 (0.542–1.484) 0.433 (0.083–2.262)	0.672 0.321	574.046
Dominant	AA + AC vs. CC	0.847 (0.520–1.377)	0.502	572.806
Recessive	AA vs. CC + AC	0.441 (0.085–2.302)	0.332	572.226
Overdominant	AC vs. CC + AA	0.912 (0.552–1.506)	0.718	573.128
Additive	A	0.824 (0.534–1.270)	0.380	572.480
*IKBKG* rs2472394				
Codominant	AC vs. CC AA vs. CC	0.861 (0.492–1.505) 0.132 (0.016–1.070)	0.599 0.058	569.431
Dominant	AA + AC vs. CC	0.715 (0.420–1.217)	0.217	571.712
Recessive	AA vs. CC + AC	0.135 (0.017–1.092)	0.060	567.708
Overdominant	AC vs. CC + AA	0.894 (0.512–1.562)	0.694	573.103
Additive	A	0.665 (0.421–1.051)	0.081	570.085

OR—odds ratio; CI—confidence interval; *p*—significance level, Bonferroni-corrected significance level *p* = 0.05/3; AIC—Akaike information criteria.

**Table 7:** Binomial logistic regression analysis of exudative AMD and control groups in females.

Model	Genotype/Allele	OR * (95% CI)	*p*-Value	AIC
*TAB2* rs237025				
Codominant	AG vs. GG AA vs. GG	1.475 (0.922–2.359) 1.826 (1.003–3.324)	0.105 **0.049**	549.184
Dominant	AA + AG vs. GG	1.561 (0.998–2.442)	0.051	547.786
Recessive	AA vs. GG + AG	1.420 (0.848–2.377)	0.182	549.832
Overdominant	AG vs. GG + AA	1.158 (0.775–1.731)	0.474	551.112
Additive	A	1.364 (1.014–1.835)	**0.040**	547.362
*IKBKB* rs13278372				
Codominant	AC vs. CC AA vs. CC	1.022 (0.618–1.690) 0.841 (0.238–2.972)	0.933 0.788	553.543
Dominant	AA + AC vs. CC	1.000 (0.618–1.616)	0.998	551.626
Recessive	AA vs. CC + AC	0.837 (0.238–2.944)	0.782	551.550
Overdominant	AC vs. CC + AA	1.028 (0.623–1.696)	0.914	551.614
Additive	A	0.981 (0.651–1.479)	0.927	551.618
*IKBKG* rs2472394				
Codominant	AC vs. CC AA vs. CC	1.137 (0.648–1.997) 0.259 (0.072–0.933)	0.655 **0.039**	548.707
Dominant	AA + AC vs. CC	0.907 (0.538–1.529)	0.714	551.491
Recessive	AA vs. CC + AC	0.254 (0.071–0.913)	**0.036**	544.907
Overdominant	AC vs. CC + AA	1.185 (0.677–2.073)	0.552	551.272
Additive	A	0.793 (0.517–1.217)	0.288	550.500

* OR adjusted for age in exudative AMD group; OR—odds ratio; CI—confidence interval; *p*—significance level, Bonferroni-corrected significance level *p* = 0.05/3; AIC—Akaike information criteria; *p* values in bold are statistically significant.

**Table 8:** Genotype and allele frequencies of *TAB2* rs237025, *IKBKB* rs13278372, and *IKBKG* rs2472394 in early and exudative AMD and control groups between males.

Genotype/Allele	Groups			*p*-Value *	*p*-Value **
	Control, n (%)	Early AMD, n (%)	Exudative AMD, n (%)		
*TAB2* rs237025					
GG	42 (35.3)	24 (27.9)	39 (32.5)	0.530	0.706
AG	58 (48.7)	46 (53.5)	57 (47.5)		
AA	19 (16.0)	16 (18.6)	24 (20.0)		
G	142 (59.7)	94 (54.7)	135 (56.3)	0.311	0.450
A	96 (40.3)	78 (45.3)	105 (43.7)		
*IKBKB* rs13278372					
CC	102 (85.7)	67 (77.9)	100 (83.3)	0.218	0.569
AC	17 (14.3)	18 (20.9)	19 (15.8)		
AA	0 (0)	1 (1.2)	1 (0.8)		
C	221 (92.9)	152 (88.4)	219 (91.3)	0.118	0.516
A	17 (7.1)	20 (12.6)	21 (8.7)		
*IKBKG* rs2472394					
CC	106 (89.1)	83 (96.5)	113 (94.2)	0.087	0.205
AC	5 (4.2)	0 (0)	1 (0.8)		
AA	8 (6.7)	3 (3.5)	6 (5.0)		
C	217 (91.2)	166 (96.5)	227 (94.6)	**0.032**	0.147
A	21 (8.8)	6 (3.5)	13 (5.4)		

*p*—significance level and Bonferroni-corrected significance level when *p* = 0.05/3; AMD—age-related macular degeneration; * early AMD vs. control group; ** exudative AMD vs. control group; *p* values in bold are statistically significant.

**Table 9:** Binomial logistic regression analysis of early AMD and control groups in males.

Model	Genotype/Allele	OR (95% CI)	*p*-Value	AIC
*TAB2* rs237025				
Codominant	AG vs. GG AA vs. GG	1.388 (0.737–2.615) 1.474 (0.641–3.390)	0.310 0.362	281.573
Dominant	AA + AG vs. GG	1.409 (0.771–2.575)	0.265	279.597
Recessive	AA vs. GG + AG	1.203 (0.579–2.501)	0.621	280.611
Overdominant	AG vs. GG + AA	1.209 (0.694–2.108)	0.502	280.404
Additive	A	1.238 (0.825–1.858)	0.302	279.787
*IKBKB* rs13278372				
Codominant	AC vs. CC AA vs. CC	1.612 (0.776–3.348) -	0.201 -	279.474
Dominant	AA + AC vs. CC	1.701 (0.825–3.507)	0.150	278.778
Recessive	AA vs. CC + AC	-	-	-
Overdominant	AC vs. CC + AA	1.588 (0.765–3.297)	0.214	279.316
Additive	A	1.754 (0.873–3.523)	0.114	278.337
*IKBKG* rs2472394				
Codominant	AC vs. CC AA vs. CC	- 0.479 (0.123–1.862)	- 0.288	276.095
Dominant	AA + AC vs. CC	0.295 (0.081–1.068)	0.063	276.646
Recessive	AA vs. CC + AC	0.502 (0.129–1.948)	0.319	279.777
Overdominant	AC vs. CC + AA	-	-	-
Additive	A	0.575 (0.284–1.163)	0.123	278.081

OR—odds ratio; CI—confidence interval; *p*—significance level, Bonferroni-corrected significance level *p* = 0.05/3; AIC—Akaike information criteria.

**Table 10:** Binomial logistic regression analysis of exudative AMD and control groups in males.

Model	Genotype/Allele	OR * (95% CI)	*p*-Value	AIC
*TRADD* rs868213				
Codominant	AG vs. AA GG vs. AA	0.371 (0.177–0.776) -	**0.009** -	320.714
Dominant	GG + AG vs. AA	0.341 (0.164–0.709)	**0.004**	320.312
Recessive	GG vs. AA + AG	-	-	-
Overdominant	AG vs. AA + GG	0.381 (0.182–0.798)	**0.011**	322.319
Additive	G	0.344 (0.170–0.697)	**0.003**	319.347
*TAB2* rs237025				
Codominant	AG vs. GG AA vs. GG	1.094 (0.616–1.942) 1.381 (0.654–2.918)	0.759 0.397	330.592
Dominant	AA + AG vs. GG	1.166 (0.679–2.003)	0.578	329.009
Recessive	AA vs. GG + AG	1.310 (0.672–2.555)	0.428	328.687
Overdominant	AG vs. GG + AA	0.978 (0.586–1.632)	0.932	329.312
Additive	A	1.162 (0.807–1.673)	0.419	328.664
*IKBKB* rs13278372				
Codominant	AC vs. CC AA vs. CC	1.118 (0.547–2.282) -	0.760 -	329.589
Dominant	AA + AC vs. CC	1.186 (0.585–2.403)	0.636	329.095
Recessive	AA vs. CC + AC	-	-	-
Overdominant	AC vs. CC + AA	1.106 (0.542–2.257)	0.782	329.243
Additive	A	1.250 (0.634–2.465)	0.519	328.901
*IKBKG* rs2472394				
Codominant	AC vs. CC AA vs. CC	0.153 (0.017–1.364) 0.729 (0.242–2.196)	0.093 0.574	327.207
Dominant	AA + AC vs. CC	0.488 (0.186–1.282)	0.145	327.101
Recessive	AA vs. CC + AC	0.758 (0.252–2.278)	0.058	329.073
Overdominant	AC vs. CC + AA	0.156 (0.017–1.387)	0.096	325.526
Additive	A	0.746 (0.434–1.280)	0.287	328.150

* OR adjusted for age in exudative AMD group; OR—odds ratio; CI—confidence interval; *p*—significance level, Bonferroni-corrected significance level *p* = 0.05/3; AIC—Akaike information criteria; *p* values in bold are statistically significant.

**Table 11:** Demographic and clinical parameters.

Characteristic		Non-Responders n = 22	Responders n = 86	*p*-Value
Gender	Females, n (%)	14 (63.6)	58 (67.4)	0.735 *
	Males, n (%)	8 (36.4)	28 (32.6)	
Age years; mean (SD)		75.91 (7.64)	77.12 (8.28)	0.537 **
Response parameter				
VA, median (IQR)				
Before treatment		0.18 (0.32) ^1^	0.35 (0.25) ^3^	0.074 ***
After 3 months		0.21 (0.37) ^1^	0.30 (0.33) ^3^	0.092 ***
After 6 months		0.21 (0.29) ^1^	0.35 (0.35) ^3^	0.029 ***
CMT (μm), median (IQR)				
Before treatment		410 (174.5) ^2^	298 (101.75) ^4^	<0.001 ***
After 3 months		282 (107.5) ^2^	262 (84.0) ^4^	0.411 ***
After 6 months		279 (109.25) ^2^	273 (87.0) ^4^	0.771 ***

*p*—significance level, significant when *p* < 0.05; IQR—interquartile range; SD—standard deviation; VA—visual acuity; CRT—central macular thickness; * Pearson’s chi-squared test; ** Student’s *t* test; *** Mann–Whitney U test; ^1^ Friedman test, *p* = 0.695; ^2^ Friedman test, *p* = 0.002; ^3^ Friedman test, *p* = 0.048; ^4^ Friedman test, *p* < 0.001; *p* values in bold are statistically significant.

**Table 12:** Associations between *TAB2* rs237025, *IKBKB* rs13278372, and treatment effectiveness.

Genetic Model	Genotype/Allele	Non-Responders n = 22	Responders n = 86	OR (95% CI)	*p*-Value	AIC
*TAB2* rs237025						
Codominant	AG vs. GG AA vs. GG	12 (54.5) 3 (13.6)	44 (51.2) 25 (29.1)	1.510 (0.509–4.478) 3.431 (0.776–15.168)	0.458 0.104	110.236
Dominant	AA + AG vs. GG	15 (68.1)	69 (80.2)	1.894 (0.668–5.372)	0.230	109.804
Recessive	AA vs. GG + AG	7 (31.8)	17 (19.8)	2.596 (0.705–9.558)	0.152	108.777
Overdominant	AG vs. GG + AA	12 (54.5)	44 (51.2)	0.873 (0.341–2.234)	0.777	111.106
Additive	A	15 (68.1)	69 (80.3)	1.794 (0.892–3.610)	0.101	108.404
*IKBKB* rs13278372						
Codominant	AC vs. CC AA vs. CC	4 (18.2) 2 (9.1)	12 (14.0) -	0.649 (0.185–2.273) -	0.499 -	106.236
Dominant	AA + AC vs. CC	6 (27.3)	12 (14.0)	0.432 (0.141–1.324)	0.142	109.156
Recessive	AA vs. CC + AC	2 (9.1)	-	-	-	-
Overdominant	AC vs. CC + AA	4 (18.2)	12 (14.0)	0.730 (0.210–2.530)	0.619	110.949
Additive	A	6 (27.3)	12 (14.0)	0.347 (0.145–0.961)	**0.041**	107.153

OR—odds ratio; CI—confidence interval; *p*—significance level, significant when *p* = 0.05; AIC—Akaike information criteria; *p* values in bold are statistically significant.

## Data Availability

The datasets used and/or analyzed during the current study are available from the corresponding author on reasonable request.
